# Maintaining reading experience continuity across e-book revisions

**DOI:** 10.1186/s41039-018-0093-9

**Published:** 2018-12-20

**Authors:** Christopher Yang, Brendan Flanagan, Gokhan Akcapinar, Hiroaki Ogata

**Affiliations:** 1 Graduate School of Informatics, Kyoto University, 36-1 Yoshida-Honmachi, Sakyo-ku, Kyoto, 606-8501 Japan; 2 Academic Center for Computing and Media Studies, Kyoto University, Yoshida-Nihonmatsu, Sakyo-ku, Kyoto, 606-8501 Japan; 3 Department of Computer Education and Instructional Technology, Hacettepe University, Ankara 06800, Turkey

**Keywords:** E-book reader, Learning footprint, Page similarity comparison, Image similarity processing, Text similarity processing

## Abstract

E-book reader supports users to create digital learning footprints in many forms like highlighting sentences or taking memos. Nowadays, it also allows an instructor to update their e-books in the e-book reader. However, e-book users often face problems when trying to find learning footprints they made in a new version e-book. Thus, users’ reading experience continuity across e-book revisions is hard to be maintained and seems to become a shortcoming within the e-book system. In this paper, in order to maintain users’ reading experience continuity, we deal with the transfer of learning footprints such as a marker, memo, and bookmark across e-book revisions on an e-book reader in a coursework scenario. We first give introduction and related works to demonstrate how researchers dedicated on the problem mentioned in this paper and page similarity comparison. Then, we compare three page similarity comparison methods using similarity computing models to compute page pairwise similarity in *image* level, *text* level, and *image & text* level. In the analysis, for each level, we analyze the performance of transferring learning footprint across e-book revisions and also the optimal threshold for similar page determination. After that, we give the analysis results to show the performances of three methods in *image* level, *text* level, and *image & text* level, and then, the error analysis is presented to specify the error types that occur in the results. We then propose page *image & text* similarity comparison as the optimal method to automatically transfer learning footprints across e-book revisions based on the analysis results and error analysis among three compared methods. Finally, the discussion and conclusions are shown in the end of this paper.

## Introduction

Recently, web-based educational applications are expected to be able to adapt users with very different background, prior knowledge of the subject, and learning goals. According to the demands, an e-book reader is one of the most prominent varieties of web-based educational systems (Brusilovsky et al. 1998). In e-book readers, the uses of annotation technique in the electronic document environment have rapidly widely treated more and more advantageous (Brush et al. 2001; Cadiz et al. 2000). An e-book reader often allows users to create learning footprints in many forms.

Furthermore, traditional textbook usually not allows teachers to update their learning materials, so the versions of learning materials are hard to be distributed. However, nowadays, e-book reading system can overcome this problem, allowing teachers to update their learning material freely. In addition, users can also create their learning footprints on e-book reading system such as taking memo or drawing highlight in any location of the interface. However, there is still a problem that exist on e-book reading system. Since e-book systems allow teachers to update learning materials in anytime, we may have to concentrate on the transfer of the created learning footprints across e-book revisions. Otherwise, e-book users’ reading experience continuity is difficult to be maintained since users cannot easily find their learning footprints in the new revision of the learning material. This problem has been mentioned as an annotation reflowing or annotation repositioning problem, such techniques have also been considered in several researches (Bargeron and Moscovich 2003; Golovchinsky and Denoue 2002; Shilman and Wei 2004). According to the problems above, this paper will summarize these problems to a learning footprint transferring problem. The objective of this paper is to automatically transfer learning footprints across e-book revisions in the BookRoll, which is an e-book reader, and be able to support many kinds of interaction between the users and system, including taking memos and highlighting text (Ogata et al. 2015; Flanagan and Ogata 2017).

**Fig. 1:**
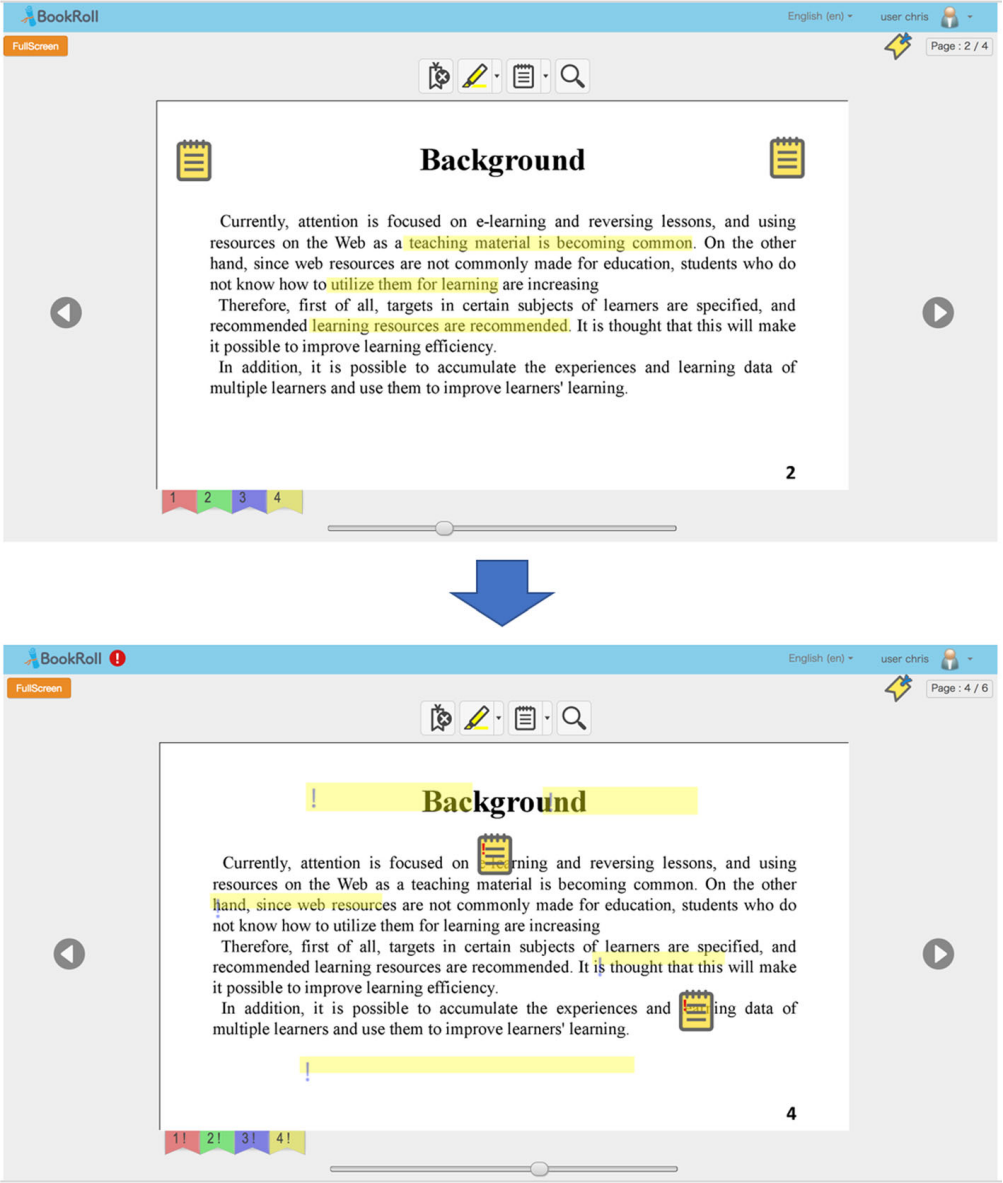
Learning footprint transferring problem on an e-book reader

Since the BookRoll allows teachers to update their e-books frequently for the new information, several versions can be distributed in one e-book. The BookRoll is also able to record user’s learning activities; however, the learning logs can only be linked with one version in one e-book (Yang et al. 2018). As shown in Fig. 1, most of the learning footprints are still being connected with the previous revision of learning material, leading to the outcome that users need to remove these incorrect learning footprints by themselves and create them again. This will severely affect users’ reading experience when using e-book system for learning. Thus, in order to maintain users’ reading experience continuity, in this paper, we compare three page similarity comparison methods to find similar pages between different revisions of one learning material since we aim to transfer learning footprints across different pages, instead of finding a new location in the same page. Learning footprints will either be transferred to the pages in the new revision or be removed from learning material depending on the pairwise page similarity and optimal threshold which will be explained in the “Methods” section. Then, we propose the optimal method for learning footprint transferring based on the analysis results.

In addition, we aim to process different kind of page contents in slide-based e-book, so we categorize page contents into three types. Pages that contain only text contents are categorized to *text* page, pages that contain only image contents are categorized to *image* page, and pages that contain both text and image contents are categorized to *image & text* page.

## Related works

### Learning footprint transferring

When the underlying document changes, an annotation may need to adapt in response. By adapting the annotation, it retains its meaning and value (Sutherland et al. 2016). Several researchers have dedicated on annotation repositioning problem using anchoring mode at a more granular level than a whole page in a modified version web-based document. Some of the articles used a line bounding box (Priest and Plimmer 2006; Chen and Plimmer 2007); one used bounding box based on HTML elements (Plimmer et al. 2010); and two used word level bounding boxes (Bargeron and Moscovich 2003; Golovchinsky and Denoue 2002). They mentioned annotations will become orphan in a document when the on-line documents changed, since these annotations lost the link to their proper location within the document. They aimed to find a proper location to anchor and reposition annotations on the modified new version document. In other words, they were also trying to transfer digital footprints from old version web-based document to a new version document. Among these articles dedicating on digital annotation repositioning problem, none of them tried to reposition annotations by page similarity comparison. In this paper, we focus on learning footprint transferring by page similarity comparison in the context of e-book, so we consider more on how accurately these methods can successfully transfer learning footprints between pages in different revisions of slide-based learning material, instead of the location of learning footprints on a specific page. However, we will take the incorrect location of learning footprints into account as a kind of error when testing the performances of methods.

### Page similarity comparison

Similarity comparison has been widely used in many research domains like object classification and document clustering. The process of similarity comparison and ranking makes the similarity measure more robust acting as a filter and eliminating the noise contained in the values of the quantitative properties (Dinu and Ionescu 2012).

Content similarity at both book level and page level have been compared, and the relationship between books are clustered and classified (Spasojevic and Poncin 2011). In this paper, the method they used to preprocess text contents in a corpus and compute page pairwise similarity is word N-Gram model and Jaccard similarity measure. The first difference between our research and theirs is the research purpose. They tried to compare book-to-book similarity and page-to-page similarity for the relationship classifying, and our purpose is to compare page-to-page pairwise similarity across e-book revisions for learning footprint transferring. The second difference is the similarity measure. They proposed Locally Sensitive Hashing (LSH) which can be used to compute set similarity during a corpus also known as Jaccard similarity. In this paper, we compare two similarity measure cosine similarity and Jaccard similarity for the representation of page similarity and propose cosine similarity as a better similarity measure since it can perform faster than Jaccard similarity. Besides, page similarity was compared for the detection of web phishing (Sanglerdsinlapachai and Rungsawang 2010). In this paper, they tried to find a term frequency matrix for pages and used cosine similarity model to represent the similarity between pages. Their experiments were focused on comparing machine learning techniques and thresholds based on page similarity comparison and the performances represented by F-measure. In our research, instead of comparing web-based pages, we try to compare slide-based pages for learning footprints transferring across e-book revisions.

As shown above, many researchers have dedicated on learning footprints transferring problem and page similarity comparison no matter in book level, page level, or web-based page level and further applied the similarities to other research field. Nevertheless, none of them focused on transferring learning footprints across e-book revisions based on page similarity comparison. In this paper, we compared three different methods based on page similarity comparison and propose the optimal one as a solution for learning footprint transferring problem in an e-book reader.

### Research questions

In order to transfer learning footprints from old version e-book to new version e-book, we compare page similarities across e-book revisions in an e-book reader. Furthermore, it is not easy to determine one page in new version e-book is similar and the other pages are not similar to the source page in old version e-book. To determine similar pages across e-book revisions, it is important to find an optimal threshold for the determination of similar pages across e-book revisions. Thus, in this paper, we give two research questions:

1. How accurately can learning footprints be transferred across e-book revisions when comparing page similarity by *image*, *text*, and *image & text*?
2. What is the optimal method to automatically transfer learning footprints across e-book revisions?

## Methods

This paper uses the BookRoll digital learning material reading system which can offer many types of interaction between users and system as our e-book reader. Users can use marker function to highlight sections of learning materials in yellow for the sections that were not understood, or red for import sections. Memo function can also be created at any pages with the specific section of the page. Users can also use bookmark function to mark any pages. Currently, e-book contents can be uploaded to the BookRoll in PDF format and be able to support a large scale of devices as it can be accessed through a standard web browser. In this paper, we implement the methods that to be compared to a slide-based e-book which is being used in the BookRoll.

**Fig. 2:**
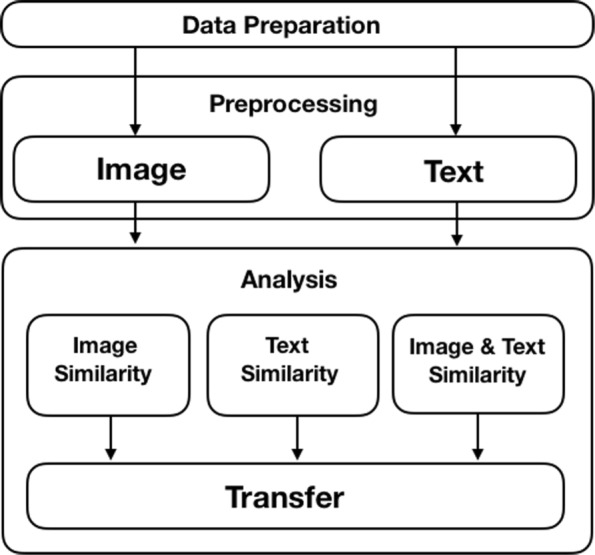
Method overview

Figure 2 shows the structure of three methods that to be compared; in data preparation, we prepare a slide-based e-book on the BookRoll to be implemented to the following three methods. Each slide-based page is categorized to one type of page contents as shown in Table 1. For image preprocessing, we use image processing models Normalized Mean Square Error (NMSE) and Structural Similarity (SSIM) to preprocess image contents and represent page image similarities between old version e-book and new version e-book. In text preprocessing, we use vector space modeling technique TFIDF weighting method to preprocess English text contents and then apply this model to cosine similarity and Jaccard similarity coefficient measure to represent page text similarities across e-book revisions. Then, we analyze the optimal threshold for similar page determination and evaluate the performances of learning footprint transferring for each method.

**Table 1:** Type of page content and distribution of learning footprint

Type	Page	Marker		Memo		Bookmark		Total	
		T	N-T	T	N-T	T	N-T	T	N-T
Image	20	10	10	10	10	10	10	30	30
Text	20	10	10	10	10	10	10	30	30
Image & text	20	10	10	10	10	10	10	30	30
Total	60	30	30	30	30	30	30	90	90

### Data preparation

**Fig. 3:**
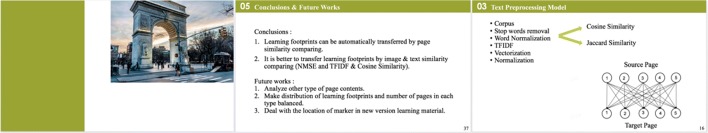
Three types of e-book slides

**Fig. 4:**
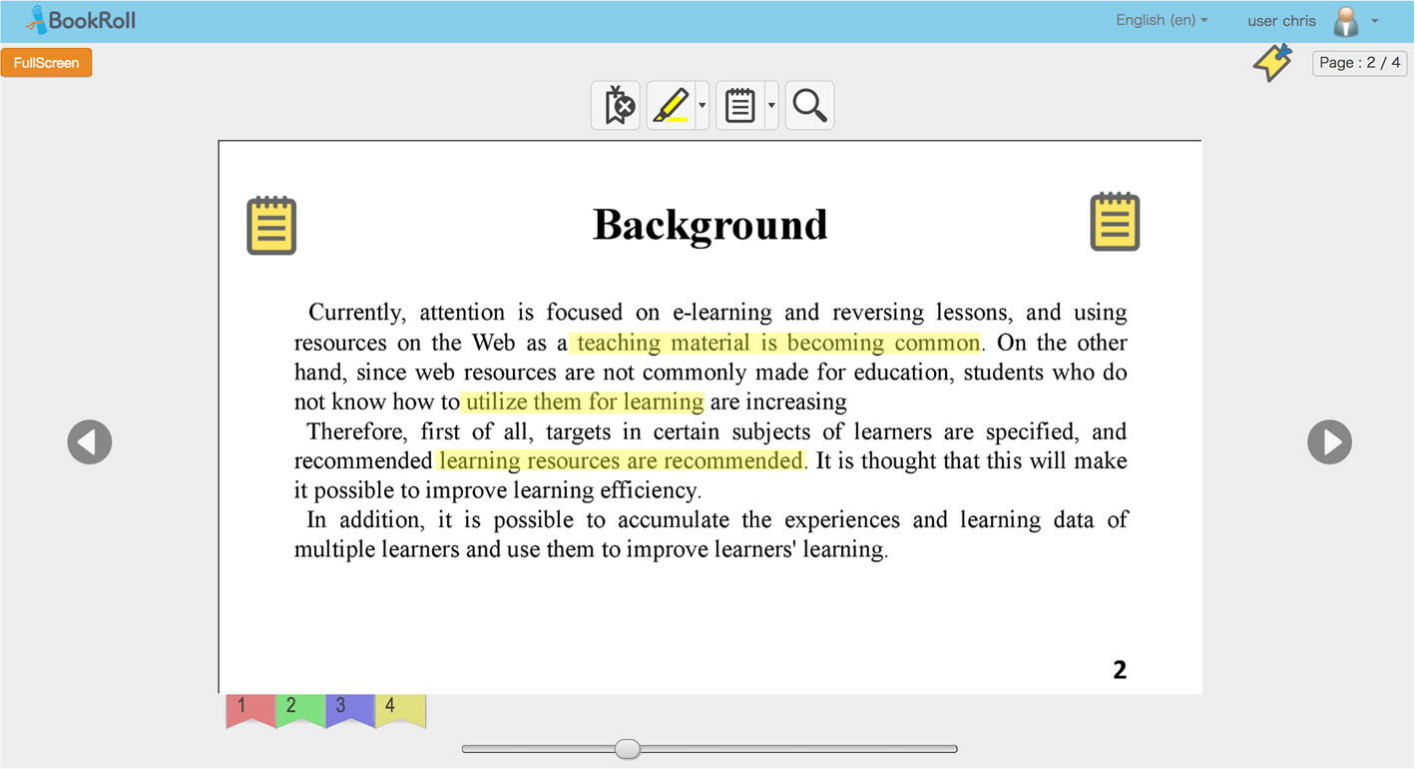
Learning footprints on the BookRoll

We prepared an old version e-book with 60 common type of English page slide contents based on an original real lecture learning material as the old version e-book, and another 30 page slide contents as the new version e-book. The original e-book contains 79 pages, we eliminated pages that contain other language text content and pages that only contain one or two lines and also pages that contain too many symbols, numbers, or equations. As shown in Table 1, this old version e-book contains 20 *image* pages, 20 *text* pages, and 20 *image & text* pages. The example of each type of page content is given in Fig. 3. We extracted page image contents and a corpus for page text contents and prepared 3 learning footprints (one marker, one memo, one bookmark) on each page. Totally, 180 learning footprints are prepared to be implemented to the following preprocessing and learning footprint transferring methods. Figure 4 shows three types of learning footprints on the BookRoll. The distribution of learning footprints is fully balanced and shown in Table 1.

We then create gold-standard data set for these learning footprints, each learning footprint will be categorized to *Transfer* class (T) or *Non-Transfer* class (N-T) as shown in Table 1. Learning footprints that are categorized to *Transfer* class are determined as they need to be transferred to the target page in new version e-book, and then, we further define the target page for these learning footprints. On the other hand, learning footprints that are categorized to *Non-Transfer* class are determined as they should not be transferred to new version e-book. In this paper, we define 50% of the *image* pages, *text* pages, and *image & text* pages in old version e-book are not exist in the new version e-book, so the learning footprints on those pages are categorized to *Non-Transfer* class as they should not be transferred to the new version e-book. In the following analysis, we evaluate the performance of learning footprints transferring in each method based on this gold-standard data set.

### Preprocessing

#### Image preprocessing

In order to compare page similarity in image level, in this paper, we compare two image processing quality metrics, Normalized Mean Square Error (NMSE), and Structural Similarity (SSIM), which are widely used for image quality assessment and image distortion quantification (Kumar et al. 2014; Wang et al. 2004; Wang and Bovik 2009).

Mean square error is computed by averaging the squared intensity differences between image pixels in two images that to be compared (Wang et al. 2004). In order to convert those distance values between source pages and target pages to similarities, we then normalize these values to the range of [0, 1] by dividing the original value by the maximum value among those distances. The higher a similarity is, the similar the two pages are.

In SSIM, typical strategy to preprocess images that to be compared is to eliminate distortions from images. First, the distorted image signals are scaled and aligned. Second, these signals will be transformed into a color space that is more appropriate for Human Visual System (HVS). Third, image quality assessment metrics will be converted to normalized digital values taking luminance, contrast, and structure comparison measures into account. These values are combined together to form local SSIM (Wang et al. 2004) and then be used to represent image similarity between source page and target page in old version e-book and new version e-book, respectively, in this paper. The higher a similarity is, the similar the two pages are.

#### Text preprocessing

In order to compare page similarity in text level, we preprocess page English text contents on the prepared corpus in three steps and generate two models to be compared using cosine similarity measure and Jaccard similarity measure. The first step is tokenization and stop words removal. We tokenize strings and giving an integer id for each possible token, for instance by using white spaces and punctuation as token separators. In stop words removal, we remove English words such as “the,” “am,” and “their” which do not influence the semantics of the review. The second step is word normalization and term counting. Since several previous researches have mentioned and proved that lemmatization can help increase the accuracy of the classification task and opinion mining task in nature language processing for English documents and be able to perform better on clustering of text documents when comparing with stemming (Gottipati et al. 2018; Korenius et al. 2004); in this paper, we use lemmatization as our word normalization technique. We then count the term frequency to represent the occurrences of terms in each document in the corpus.

The third step is vector space modeling. In this step, based on the term frequency matrix, we use vectorized TFIDF weighting method to compute the weight of each term on their document and represent this matrix on the vector space. The details of TFIDF weighting methods have been described in several previous studies (Wu et al. 2008; Salton and Buckley 1988).

We apply this TFIDF model to cosine similarity measure and Jaccard similarity coefficient measure for page text similarity representation. Cosine similarity is a term-based similarity measure baseline of similarity between two vectors of an inner product space that measures the cosine of the angle between them (Gomaa and Fahmy 2013). It has been widely used in several text semantic analysis tasks in Landauer and Dumais (1997); Mihalcea et al. (2006); Cheng et al. (2008); Susanti et al. (2017). Jaccard similarity coefficient (Roussinov and Zhao 2003) is a statistical measure of the extent of overlapping between two vectors. It is defined as the size of the intersection divided by the size of the union of the vector dimension sets (Cheng et al. 2008). In this paper, cosine similarity measure and Jaccard similarity coefficient measure are used to represent the text similarity between every two page text contents in old version e-book and new version e-book, which can also be considered as two vectors in the vector space generated by TFIDF model. The higher a similarity is, the similar the two pages are. In addition, cosine similarity and Jaccard similarity can be seen as a method of normalizing document length during page similarity comparison. The image preprocessing and text preprocessing algorithms we use were developed in Python scikit-learn library (Pedregosa et al. 2011).

### Analysis

In this analysis, for each method, we analyze the optimal threshold and evaluate the performance of learning footprint transferring according to the tested threshold and gold-standard data set. We compare similarities between two different revisions of learning material. Thus, in this paper, there are 30-page similarities for each page in old revision since we created a 30-page new revision of learning material as shown in Table 1, and they will be compared with the tested threshold individually. Each threshold value produces a performance point in the ROC space, and these points are linked through two consecutive points to produce a ROC curve. The need of threshold when assessing model performance using the indices derived from the confusion matrix was emphasized in several researches (Liu et al. 2005; Manel et al. 2001). In this paper, an optimal similarity threshold is an important factor for the determination of similar pages between different revisions of learning material and need to be objectively decided. For instance, learning footprints that relate to new revision of learning material could be incorrectly removed if the similarity threshold is set too high. In contrast, learning footprints that do not relate to the new revision of learning material could be incorrectly transferred if the similarity threshold is set too low.

#### Method 1: page image similarity comparison

In *image* level analysis, we test threshold in the rage of [0, 1], gradually adding it by 0.05, so overall, we compare 42 performances of learning footprint transferring and choose the best one among them since we compare two different image preprocessing models, NMSE and SSIM. For each tested threshold, if the image similarity between source page and one page in new version e-book is higher than the tested threshold and meanwhile it is the similarity that most close to the tested threshold among all the similarities, learning footprints on that page will be transferred to its target page in the new revision of the learning material. On the other hand, if all the image similarities between source page and other pages in new version e-book are lower than the tested threshold, learning footprints on that page will be removed from the BookRoll.

#### Method 2: page text similarity comparison

In *text* level analysis, we analyze and compare two different text similarity representation models cosine similarity and Jaccard similarity. We compare 42 performances according to the tested threshold and gold-standard data set, then choose the best one within them. Similar to *method 1*, for each tested threshold from 0 to 1, if the text similarity between source page and target page is higher than the tested threshold and meanwhile it is the similarity that most close to the tested threshold, learning footprints on that page will be transferred to its target page in the new revision of learning material. On the other hand, if all the text similarities between source page and other pages in new version e-book are lower than the tested threshold, learning footprints on that page will be removed from the BookRoll.

#### Method 3: page image & text similarity comparison

In *image & text* level analysis, we choose the better image proprocessing model and text preprocessing model according to the thresholds and performances we tested in *method 2* and *method 3*. First, we compare page image similarity and regard pages with higher than the optimal similarity threshold as the source page and target page. If the target pages in new version e-book can be found, learning footprints will be transferred from source page in old version e-book to its target page in new revision of learning material. For the rest pages in old version e-book, they will be analyzed by page text similarity comparison to look for other target pages in the new revision of the learning material and transfer learning footprints. If neither image similarity comparison nor text similarity comparison method can find the target page for individual source page, then learning footprints on this source page will be removed from the BookRoll. In the next section, we give the results of all the performances above represented by confusion matrix in *image* level, *text* level, and *image & text* level.

#### Evaluation

In statistical analysis of binary classification, the F-measure score is a measure of a classification accuracy derived from confusion matrix which consisted of True-Positive (TP), True-Negative (TN), False-Positive (FP), and False-Negative (FN). The F-measure quantifies the balance between precision and recall, with a larger value reflecting a better result. TP and FP derived from confusion matrix denote the numbers of learning footprints transferred correctly and incorrectly, respectively. Accordingly, TN and FN derived from confusion matrix denote the numbers of learning footprints removed correctly and incorrectly, respectively. The four indicators (TP, FP, TN, FN) are used to measure the accuracy of learning footprints transferring methods. The equations are given in (1), (2), and (3). For each method, we give the performance, distribution of successfully transferred learning footprints and error analysis. In the error analysis, we investigate the error type and distribution of errors. We also compared CPU time for each analysis. We analyzed the compared methods and evaluate the performances on the environment with processor 1.8 GHz, Intel Core i5, and 8 GB RAM.

1 $$ \text{Precision} = \frac{\text{TP}}{\text{TP + FP}}  $$

2 $$ \text{Recall} = \frac{\text{TP}}{\text{TP + FN}}  $$

3 $$ \text{F}-\text{measure} = \frac{2 \cdot \text{precision} \cdot \text{recall}}{\text{precision + recall}}  $$

## Results

### Optimal threshold and evaluation for page *image* similarity comparison

**Fig. 5:**
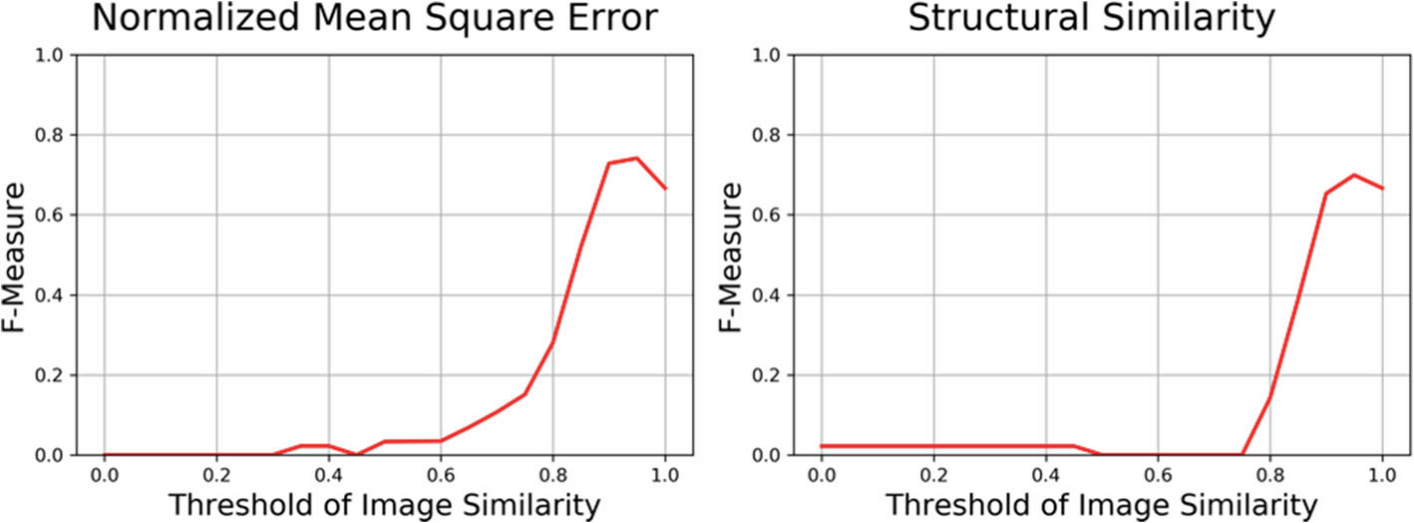
Optimal threshold for NMSE and SSIM model

As shown in Fig. 5 and Table 2, in the first model NMSE, the precision, recall, and F-measure score are 1.0, 0.589, and 0.741, respectively, when the threshold is set to 0.95. In SSIM model, the precision, recall, and F-measure score is 0.943, 0.556, and 0.699, respectively, when the threshold is set to 0.95. According to the F-measure scores and execution time, NMSE model can perform better and faster than SSIM model on learning footprints transferring; thus, we propose in page image similarity comparison, NMSE is the better model. In NMSE model, 79.4% of the learning footprints (143/180) were correctly processed, 20.6% of the learning footprints (37/180) were incorrectly processed according to the confusion matrix shown in Table 3, and the pre-defined gold-standard data set that we prepared.

**Table 2:** Model comparison between image preprocessing models

Model	Threshold	Precision	Recall	F-measure	Error rate	CPU time
NMSE	0.95	1.0	0.589	0.741	0.206	167.072 s
SSIM	0.95	0.943	0.556	0.699	0.239	2420.154 s

**Table 3:** Confusion matrix of *image* similarity comparing (NMSE)

	Gold-standard		
Prediction	Transfer	Non-transfer	Total
Transfer	53 (29.4%)	0 (0%)	53
Non-transfer	37 (20.6%)	90 (50%)	127
Total	90	90	180

### Optimal threshold and evaluation for page *text* similarity comparison

**Fig. 6:**
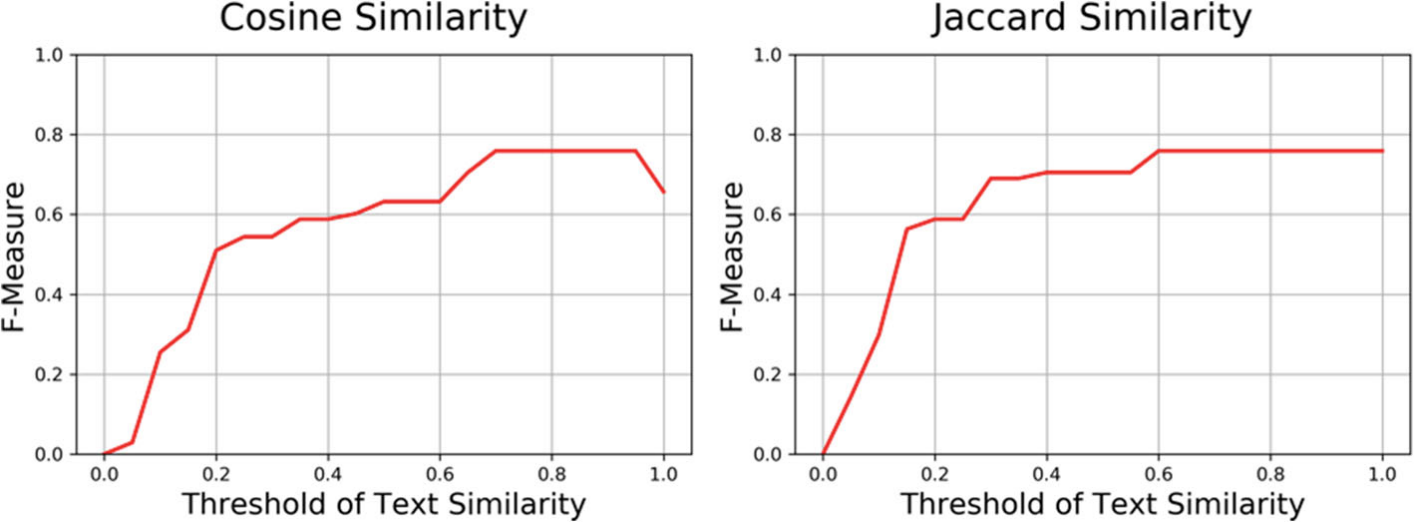
Optimal threshold for cosine similarity and Jaccard similarity models

As shown in Fig. 6 and Table 4, in the first model cosine similarity, the precision, recall, and F-measure score is 1.0, 0.611, and 0.759, respectively, when the threshold is set to the range of [0.7, 0.95]. In the second model Jaccard similarity, the precision, recall, and F-measure score is 1.0, 0.611, and 0.759, respectively, when the threshold is set to the range of [0.6, 1]. According to the F-measure scores and execution time, although the F-measure scores are the same between this two models, cosine similarity model can perform faster than Jaccard similarity model on learning footprint transferring; thus, we propose in page text similarity comparison, cosine similarity is the better model. In cosine similarity model, 80.6% of the learning footprints (145/180) were correctly processed and 19.4% of the learning footprints (35/180) were incorrectly processed according to the confusion matrix shown in Table 5 and the pre-defined gold-standard data set.

**Table 4:** Model comparison between text preprocessing models

Model	Threshold	Precision	Recall	F-measure	Error rate	CPU time
Cosine similarity	0.7–0.95	1.0	0.611	0.759	0.194	0.986 s
Jaccard similarity	0.6–1.0	1.0	0.611	0.759	0.194	2.126 s

**Table 5:** Confusion matrix of *text* similarity comparing (cosine similarity)

	Gold-standard		
Prediction	Transfer	Non-transfer	Total
Transfer	55 (30.6%)	0 (0%)	55
Non-transfer	35 (19.4%)	90 (50%)	125
Total	90	90	180

### Evaluation for page *image & text* similarity comparison

According to the results above, we choose NMSE model and threshold 0.95 image similarity to compare page image similarity then find target pages and transfer learning footprints by the *Transfer* algorithm. For the rest pages in old version e-book, we use cosine similarity model and threshold 0.7 text similarity to compare page text similarity and then find target page and transfer learning footprints. By image analyzing, this method successfully found 18 target pages and transferred 17 markers, 18 memos, and 18 bookmarks. For the rest of the pages in an old version e-book, by text analyzing, this method successfully found 7 target pages and transferred 3 markers, 7 memos, and 7 bookmarks. As shown in Table 6, the precision, recall, F-measure score, and error rate is 1.0, 0.778, 0.875, and 0.111, respectively. According to the F-measure and error rate, this method can perform better on learning footprint transferring than previous two methods, which represent only comparing image similarities or text similarities. In this method, 88.9% of the learning footprints (160/180) were correctly processed and 11.1% of the learning footprints (20/180) were incorrectly processed according to the confusion matrix shown in Table 6 and gold-standard data set. In the comparison of three methods, we propose this method is the optimal method for learning footprint transferring across e-book revisions.

**Table 6:** Confusion matrix of *image & text* similarity comparing

	Gold-standard		
Prediction	Transfer	Non-transfer	Total
Transfer	70 (38.9%)	0 (0%)	70
Non-transfer	20 (11.1%)	90 (50%)	110
Total	90	90	180

### Error analysis

In error analysis, we investigate the error type and the distribution of errors from confusion matrix. We identify 3 error types in this analysis, incorrect remove (IR), incorrect transfer to wrong page (ITP), and incorrect transfer to wrong location (ITL). Table 7 shows the error distribution of marker, for 13 errors in NMSE model, 92.3% of the errors belong to IR and 7.7% of the errors belong to ITL. For 15 errors in cosine similarity model, 66.7% of the errors belong to IR and 33.3% of the errors belong to ITL. For 10 errors in method 3, 50% of the errors belong to IR and 50% of the errors belong to ITL. Table 8 shows the error distribution of memo; for 12 errors in NMSE model, 100% of the errors belong to IR and none of the errors belong to ITP and 3. For 10 errors in cosine similarity model, 100% of the errors belong to IR and none of the errors belong to ITP and 3. For 5 errors in method 3, 100% of the errors belong to IR and none of the errors belong to ITP and 3. Table 9 shows the error distribution of bookmark; for 12 errors in NMSE model, 100% of the errors belong to IR and none of the errors belong to ITP and 3. For 10 errors in cosine similarity model, 100% of the errors belong to IR and none of the errors belong to ITP and 3. For 5 errors in method 3, 100% of the errors belong to IR and none of the errors belong to ITP and ITL.

**Table 7:** Error distribution of marker

Marker				
Level	Method	IR	ITP	ITL
Image	NMSE	12/13 (92.3%)	0/13 (0%)	1/13 (7.7%)
Text	Cosine similarity	10/15 (66.7%)	0/15 (0%)	5/15 (33.3%)
Image & text	Method 3	5/10 (50%)	0/10 (0%)	5/10 (50%)

**Table 8:** Error distribution of memo

Memo				
Level	Method	IR	ITP	ITL
Image	NMSE	12/12 (100%)	0/12 (0%)	0/12 (0%)
Text	Cosine similarity	10/10 (100%)	0/10 (0%)	0/10 (0%)
Image & text	Method 3	5/5 (100%)	0/5 (0%)	0/5 (0%)

**Table 9:** Error distribution of bookmark

Bookmark				
Level	Method	IR	ITP	ITL
Image	NMSE	12/12 (100%)	0/12 (0%)	0/12 (0%)
Text	Cosine similarity	10/10 (100%)	0/10 (0%)	0/10 (0%)
Image & text	Method 3	5/5 (100%)	0/5 (0%)	0/5 (0%)

## Discussion

In the discussion, we discuss the error distribution as shown in Tables 7, 8, and 9 and answer the two research questions mentioned in previous section. We first discuss what are the main error types occurred when comparing page similarity in different levels and adapting *Transfer* algorithm. Then, we discuss the possible reason that caused these errors along with marker, memo, and bookmark individually.

In the error distribution, we can see that IR is the most significant error type in image similarity comparison, 12 incorrect removes of marker, memo, and bookmark in NMSE model, respectively. This is because in image similarity comparison, the image similarities decreased due to the change of location of image contents and paragraphs in a page, and it would be hard to find correct target page in this situation. According to the results, this problem can be solved by text similarity comparison since it is able to find similar pages based on text similarities instead of image, and the target page can be found even the paragraphs changed.

In the error distribution, we also can see that IR is also the most significant error type in text similarity comparison, 10 incorrect removes of marker, memo, and bookmark in cosine similarity model. This is because in the new version e-book, learning footprints in 10 image pages need to be transferred but the text similarities with other pages will always be zero if a page only contains image content, which makes finding correct target page is impossible. According to the results, this problem can be solved by the proposed method, which is page *image & text* similarity comparison. Since it will first compare image similarities and find target pages that with 100% image similarities and transfer learning footprints, then compares text similarities to keep looking for target page. As shown in the results, the error rate can be decreased to 0.111 and the F-measure score can be increased to 0.875.

In *image & text* similarity comparison, the error distribution shows the main error on this method. Five incorrect removes on marker, memo, and bookmark occurred due to the change of location of image contents. Another 5 errors came from incorrect transfer to the wrong location in target page, and these 5 markers were transferred to the wrong location even the target page has been found by this method. According to the error distribution, in the proposed method, the *Transfer* algorithm needs to be improved to reduce the errors from ITL.

Furthermore, ITL occurred frequently on marker in the error distribution; however, for memo and bookmark, the error rates are 0% as they can be 100% transferred to the target pages correctly. According to the results, in this e-book, all this three methods can successfully transfer memos and bookmarks from an old version e-book to a new version e-book without an error. This is because in this e-book, memos and bookmarks are along with the slide pages, which means once the target page was found, they can be directly transferred without the concern about location in the target page, which is different from the marker in this paper.

To answer research question 1, according to the results as shown in Table 6, by comparing page similarities in *image & text* level, we obtained the highest F-measure score 0.875 and lowest error rate 11.1% when transferring learning footprints across e-book revisions compared to other two methods. Thus, this method can automatically transfer learning footprints decently and potentially be an option method when researchers focusing on similar problem mentioned in this paper.

To answer research question 2, method 3 outperformed than method 1 and method 2 as shown in Tables 2, 3, 4, 5, and 6. Thus, in this paper, we propose that the optimal method to automatically transfer learning footprints across e-book revisions is to compare page similarities in *image & text* level.

To discuss the research limitation in this paper, currently, the proposed method are only tested in slide-based learning material in e-book reading environment. For other environments like XML documents or other reading system, we do not know if this method can perform the same as we evaluated in this paper. In addition, we just analyzed three common types of page contents (text, image, image & text) from slide-based learning material as shown in Table 1. For other types of page contents, we do not know if the accuracy will be worse due to the differentiation of page contents.

## Conclusions

In this paper, we identified the problem when e-book users trying to find the learning footprints they made in the new version e-book. To address that, we compared three methods for learning footprint transferring across slide-based e-book revisions in three different levels, page *image* similarity, page *text* similarity, and page *image & text* similarity. We analyzed the optimal threshold and evaluated the performance of learning footprint transferring for each method in different levels, and the error distribution was given for the detail of errors. The performances of learning footprint transferring are presented by F-measure in a confusion matrix. According to the results, the best F-measure scores of methods 1, 2, and 3 are 0.741, 0.759, and 0.875, respectively. According to the F-measure scores, we propose method 3 which is comparing page similarity in *image & text* level, as the optimal method to automatically transfer learning footprints across e-book revisions. In this method, we used NMSE model and image similarity threshold 0.95 for image content processing, similarity representation, and similar page determination to find similar pages and transfer learning footprints. For the rest of the pages in an old version e-book, we then we used TFIDF weighting method, cosine similarity model, and text similarity threshold 0.7 for text content processing, similarity representation, and similar page determination within a slide-based e-book to find similar pages between versions and transfer learning footprints. According to the evaluation and error analysis, the location of image contents and text contents needs to be considered well; otherwise, learning footprints will be incorrectly transferred to the wrong location even the target page can be successfully found.

To our future works, in this paper, we eliminated uncommon page contents like pages that contain more than one language, pages that contain only one or two lines and pages that contain too many symbols, numbers, and equations in an e-book. In the future, we will also take other type of page contents into account since it occurs frequently in the lecture learning materials. According to the error analysis, many errors came from the incorrect location of marker; in the future, we will dedicate on how to improve the proposed method for a higher accuracy and F-measure derived from confusion matrix in context of binary classification. In addition, user studies will be conducted in the future by a series of experiment in a real coursework to further assess the effectiveness of the proposed method. Also, in user study, we will observe and quantify users’ reaction to this method. The personalized learning footprint transfer will also be focused in the future, and we will transfer learning footprints not just in page level but also in user level.
